# The global prevalence of depression, anxiety, and sleep disorder among patients coping with Post COVID-19 syndrome (long COVID): a systematic review and meta-analysis

**DOI:** 10.1186/s12888-023-05481-6

**Published:** 2024-02-06

**Authors:** Niloofar Seighali, Abolfazl Abdollahi, Arman Shafiee, Mohammad Javad Amini, Mohammad Mobin Teymouri Athar, Omid Safari, Parsa Faghfouri, Alireza Eskandari, Omid Rostaii, Amir Hossein Salehi, Hedieh Soltani, Mahsa Hosseini, Faeze Soltani Abhari, Mohammad Reza Maghsoudi, Bahar Jahanbakhshi, Mahmood Bakhtiyari

**Affiliations:** 1 Non-Communicable Diseases Research Center, Alborz University of Medical Sciences, Karaj, Iran; 2 Student Research Committee, School of Medicine, Alborz University of Medical Sciences, Karaj, Iran; 3 School of Medicine, Shahid Beheshti University of Medical Sciences, Tehran, Iran; 4 Department of Community Medicine, School of Community Medicine, Alborz University of Medical Sciences, Karaj, Iran; 5 Student research committee, Arak University of Medical Sciences, Arak, Iran

**Keywords:** Post COVID-19 syndrome, Long COVID, Depression, Anxiety, Sleep disorder, Prevalence, Systematic review, Meta-analysis

## Abstract

**Background:**

Post COVID-19 syndrome, also known as "Long COVID," is a complex and multifaceted condition that affects individuals who have recovered from SARS-CoV-2 infection. This systematic review and meta-analysis aim to comprehensively assess the global prevalence of depression, anxiety, and sleep disorder in individuals coping with Post COVID-19 syndrome.

**Methods:**

A rigorous search of electronic databases was conducted to identify original studies until 24 January 2023. The inclusion criteria comprised studies employing previously validated assessment tools for depression, anxiety, and sleep disorders, reporting prevalence rates, and encompassing patients of all age groups and geographical regions for subgroup analysis Random effects model was utilized for the meta-analysis. Meta-regression analysis was done.

**Results:**

The pooled prevalence of depression and anxiety among patients coping with Post COVID-19 syndrome was estimated to be 23% (95% CI: 20%—26%; I2 = 99.9%) based on data from 143 studies with 7,782,124 participants and 132 studies with 9,320,687 participants, respectively. The pooled prevalence of sleep disorder among these patients, derived from 27 studies with 15,362 participants, was estimated to be 45% (95% CI: 37%—53%; I2 = 98.7%). Subgroup analyses based on geographical regions and assessment scales revealed significant variations in prevalence rates. Meta-regression analysis showed significant correlations between the prevalence and total sample size of studies, the age of participants, and the percentage of male participants. Publication bias was assessed using Doi plot visualization and the Peters test, revealing a potential source of publication bias for depression (*p* = 0.0085) and sleep disorder (*p* = 0.02). However, no evidence of publication bias was found for anxiety (*p* = 0.11).

**Conclusion:**

This systematic review and meta-analysis demonstrate a considerable burden of mental health issues, including depression, anxiety, and sleep disorders, among individuals recovering from COVID-19. The findings emphasize the need for comprehensive mental health support and tailored interventions for patients experiencing persistent symptoms after COVID-19 recovery.

**Supplementary Information:**

The online version contains supplementary material available at 10.1186/s12888-023-05481-6.

## Introduction

The ongoing COVID-19 pandemic has precipitated a worldwide health catastrophe, impacting not only the physical health of those infected but also their emotional stability [1]. Post-COVID-19 syndrome, which pertains to the persistence of symptoms beyond the expected recovery period, has become a burgeoning concern and has been correlated with the emergence of mental health afflictions such as depression and anxiety [2]. According to the National Institute for Health and Care Excellence (NICE), "post-COVID-19 syndrome" (PCS) is a complex set of symptoms that emerge during or after a COVID-19 infection, lasting for a minimum of 12 weeks, and cannot be explained by alternative diagnoses. PCS often presents with various clusters of symptoms that can change over time and impact multiple systems in the body. These symptoms may overlap and may fluctuate in severity [3]. This development underscores the necessity of additional research into the correlation between post-COVID-19 syndrome and mental health, as well as the urgency of addressing the emotional needs of those affected by the virus.

Research has established that individuals with post-COVID-19 syndrome are more susceptible to depression and anxiety. Around 30 to 40% of individuals who have recovered from COVID-19 have been reported for experiencing symptoms such as anxiety, depression, sleep issues, and post-traumatic stress disorder, which is similar to what has been observed in survivors of other coronaviruses [4].

The exact mechanisms by which post-COVID-19 syndrome contributes to the development of depression and anxiety remain unclear. However, it is theorized that the physical and psychological stress caused by the illness may play a role. For instance, the extended nature of the illness and the uncertainty of its outcome can engender feelings of hopelessness and frustration. Post-COVID-19 syndrome often entails persistent physical symptoms such as fatigue, respiratory difficulties, and muscle and joint pain, which can severely impact quality of life and daily functioning [5]. Additionally, the social and economic ramifications of the pandemic may exacerbate these feelings, with many individuals facing job loss, financial insecurity, and social isolation [6]. On the other hand, mechanisms originated from the immune system might play a role. Dysregulations in the immune responses, thrombosis in cerebral microvasculature, neuroanatomical alterations, and adverse effects of drugs administered during the disease potentially predispose the patients to neuropsychiatric complications [7, 8].

Considering the high rates of mental disorders linked to post-COVID-19 syndrome, it is imperative to conduct further research to comprehend the mechanisms by which the illness may be contributing to these mental health conditions. This will facilitate healthcare providers in developing more effective interventions and treatments for those affected by post-COVID-19 syndrome.

The COVID-19 pandemic has inflicted a considerable toll on the mental health of individuals, particularly those affected by post-COVID-19 syndrome. Elucidating the unprecedented aspects of this issue provides healthcare providers with adequate knowledge to mitigate the negative impact of post-COVID-19 syndrome on mental health and enhance the quality of life for those affected by the illness. The strong association between post-COVID-19 syndrome and depression, anxiety, and sleep disturbances emphasizes the importance of further research on this subject and the necessity of evaluating the extent of this interplay. Therefore, we conducted a thorough systematic review and meta-analysis to address this issue by investigating the incidence of mental disorders among patients experiencing post-COVID-19 syndrome.

## Methods

The present systematic review was conducted based on Preferred Reporting Items for Systematic Reviews and Meta-Analyses (PRISMA) guidelines and guideline retrieved from the Cochrane Handbook for Systematic Reviews of Interventions [9, 10]. The protocol of this review was prospectively registered on PROSPERO with the following registration code: CRD42023413023.

### Search strategy

We performed a comprehensive database search in PubMed, Scopus, Embase, and Web of science up to 24 January 2023, using the search string with the combination of following keywords: post-acute covid-19 syndrome, long covid-19 syndrome, COVID-19 survivors, COVID-19 sequelae, depression, anxiety, sleep, and prevalence (Supplementary File). No limitation was implemented on our search results.

### Eligibility criteria

The inclusion criteria definition was: 1) Population: patients who were recovered from SARS-CoV-2 in their past medical history; 2) Assessment: previously validated measurements for depression, anxiety, and sleep scales; 3) Comparison: not applicable; 4) Outcome: reported the prevalence rate; 5) Type of Study: all types of original studies. There was no limitation on date and the language of the published report. Case report studies, review studies, meta-analyses, commentary studies, and letter to editor articles without any relevant data were excluded.

### Screening and data extraction

Two-step screening were performed by our reviewers. Disagreements were resolved by the third reviewer (A.S.). Data were extracted on an Excel spreadsheet. The extracted data included Author, Year, Country, Population, Total patients, Assessment scales, Cut-offs, and depression, anxiety, and sleep disorders prevalence.

### Quality assessment

Four reviewers independently evaluated the included studies using the Newcastle–Ottawa Quality Assessment [11]. The studies with 7 or more yeses are rated as “Good”, 5–6 as “Fair”, and with 4 or fewer as “Poor" quality report.

### Synthesis

We conducted a meta-analysis using a proportion random effects model to estimate the prevalence of depression, anxiety, and sleep disorder. Given the diverse study populations and methods, we used a random effects model to summarize the prevalence, presenting proportions and 95% confidence intervals (95% CI). To assess heterogeneity, we utilized the Cochrane Q-test and I2 statistic, considering I2 values higher than 75% as high levels of heterogeneity. We evaluated publication bias using Doi plot visualization and Peters test [12, 13]. Conducting a subgroup analysis, we categorized studies based on their geographical region, and assessment measure used. Additionally, we examined the moderating effect of factors such as year of publication, sample size, age, and percentage of male participants through conducting a meta-regression analysis. All statistical analyses and graphical representations were performed using STATA, R (version 4.1.3) and the meta package (version 5.5) [14].

## Results

### Characteristics

**Fig. 1:**
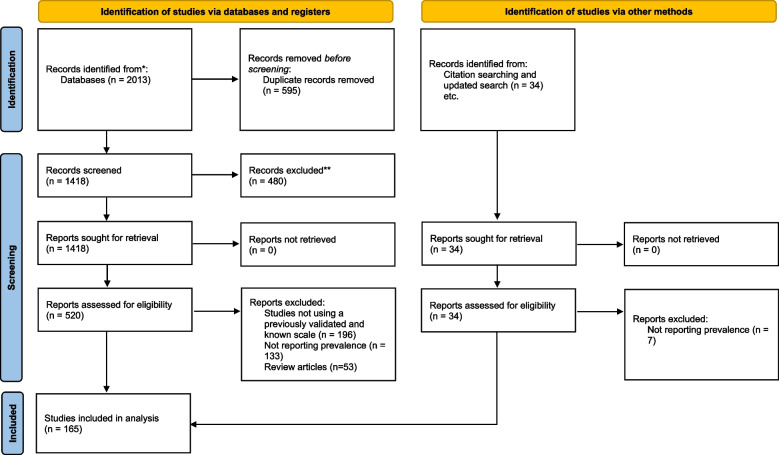
PRISMA flow diagram

**Fig. 2:**
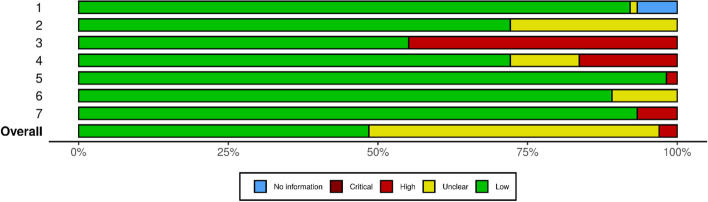
Results of quality assessment

After examining a total of 2013 records, 165 studies with 9,923,461 total patients were included in our review [15–179] (Fig. 1). Eighty of the studies were designed in cohort [87–166], 71 cross-sectional [16–86], 7 case control [167–173], 4 survey study [174, 176–178], 1 randomized clinical trial [15] and 2 secondary analysis [175, 179]. Included studies were published between 2019–2023. The most common assessment scales used for depression diagnosis were BDI, DASS, HADS, PHQ, and for anxiety were GAD, HADS-A, and for sleep disorders were PSQI. We assessed the risk of bias for each study using the quality assessment checklist based on Newcastle–Ottawa Scale. Based on the results of our quality assessment, there were 81 studies with good, 79 with fair and 5 with poor methodological quality. Detailed characteristics of each study and its quality assessment are provided in Supplementary File (Fig. 2).

### Prevalence of depression

Data from 143 studies with 7,782,124 participants reporting the relevant outcome were included. The pooled prevalence of depression among patients coping with Post COVID-19 syndrome was estimated to be 23% (95% CI: 20%—26%; I2 = 99.9%) (Supplementary Fig. 1). Subgroup analysis based on geographical regions and assessment scales showed a significant between-subgroup differences (Table 1).

**Table 1:** Results of meta-analysis

**Outcome**	**No. of studies**	**Prevalence **	**[95% Confidence interval]**	**I2**
Depression	142	23%	20% - 26%	99%
Anxiety	131	23%	20% - 26%	99%
Sleep disorder	27	45%	37% - 53%	98%

### Prevalence of anxiety

Data from 132 studies with 9,320,687 participants reporting the relevant outcome were included. The pooled prevalence of anxiety among patients coping with Post COVID-19 syndrome was estimated to be 23% (95% CI: 20%—26%; I2 = 99.9%) (Supplementary Fig. 2). Subgroup analysis based on geographical regions and assessment scales showed a significant between-subgroup differences (Table 1).

### Prevalence of sleep disorder

Data from 27 studies with 15,362 participants reporting the relevant outcome were included. The pooled prevalence of sleep disorder among patients coping with Post COVID-19 syndrome was estimated to be 45% (95% CI: 37%—53%; I2 = 98.7%) (Supplementary Fig. 3). Subgroup analysis based on geographical regions and assessment scales showed a significant between-subgroup differences (Table 1).

### Meta-regression analysis

A meta-regression analysis was performed to examine the potential moderating effects of factors such as year of publication, sample size, age, and percentage of male participants. The results of meta-regression showed significant correlation between the prevalence of 1) depression and total sample size and male percentage of the included studies; 2) anxiety and total sample size of the included studies; and 3) sleep disorder and age and male percentage of the included studies (Table 2).

**Table 2:** Results of meta-regression

	**Coefficient**	**Standard error**	***P* >|z|**	**95% CI**
**Depression**				
Year	-0.04	0.06	0.46	-0.15 to 0.07
Total	-1.36e-07	5.09e-08	**0.008**	-2.35e-07 to -3.58e-08
Age	-0.003	0.003	0.32	-0.009 to 0.003
Male (%)	-0.005	0.002	**0.02**	
**Anxiety**				
Year	0.02	0.06	0.77	-0.10 to .014
Total	-1.00e-07	5.08e-08	**0.04**	-2.00e-07 to -6.40e-10
Age	-0.001	0.003	0.66	-0.008 to 0.005
Male (%)	-0.0003	0.0009	0.72	-0.002 to 0.001
**Sleep disorder**				
Year	-0.19	0.13	0.14	-0.46 to 0.06
Total	-.00007	.00005	0.17	-0.00017 to 0.00003
Age	0.03	0.01	**0.01**	0.006 to 0.049
Male (%)	-0.011	0.005	**0.04**	-0.0229 to -0.0003

### Publication bias

**Fig. 3:**
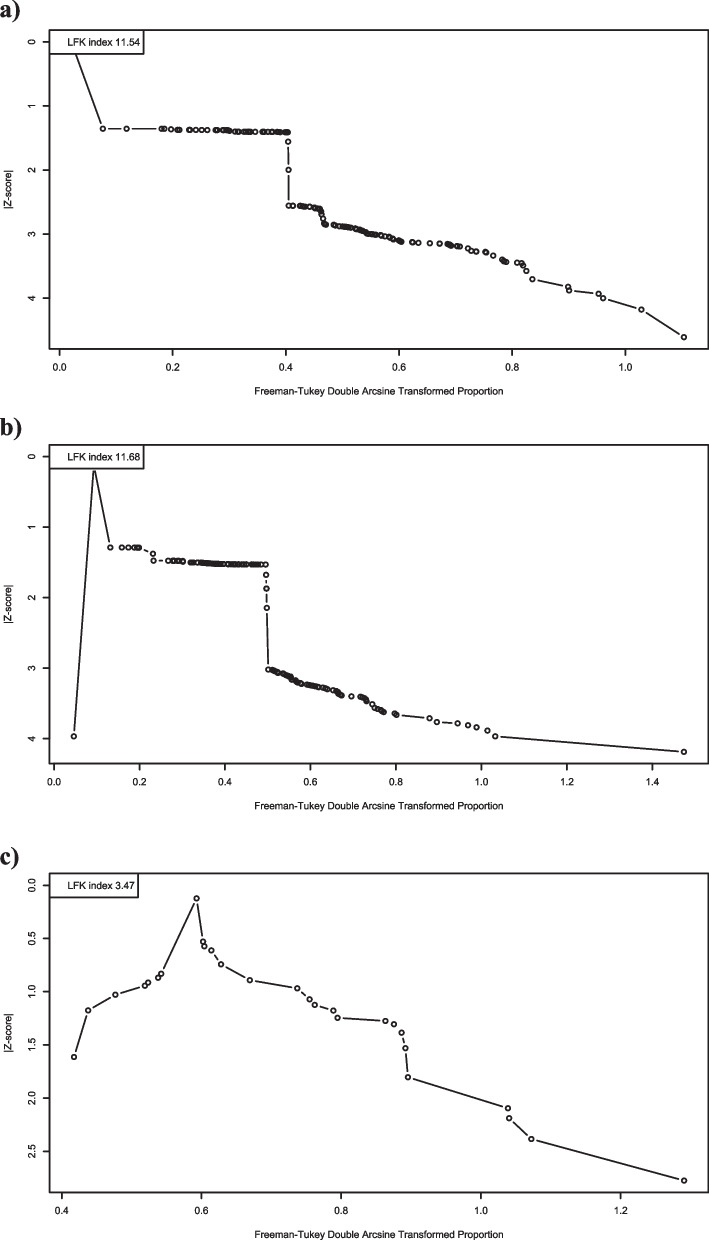
Doi plots for the prevalence of **a**) depression; **b**) anxiety; and **c**) sleep disorder

Publication bias was assessed using Doi plot visualization and Peters test. The results showed a significant source of possible publication bias based on peters test for depression (*p* = 0.0085) and sleep disorder (*p* = 0.02). There were no sources of publication bias for anxiety (*p* = 0.11). The Doi plots are presented in Fig. 3.

## Discussion

To the best of our knowledge, this systematic review and meta-analysis represents the most comprehensive and wide-ranged investigation of depression, anxiety, and sleep disorders among long COVID patients. The primary objective of this study was to provide an updated and accurate evaluation of the prevalence rates of these mental health conditions by analyzing data from 66 studies. The findings from our study indicated that COVID-19 patients have a general prevalence of 23% for depression, 23% for anxiety, and 37% for sleep disturbances.

Assessing the prevalence of mental disorders such as depression, anxiety, and sleep disturbance among individuals with the post-COVID syndrome is crucial for comprehending the complete impact of the COVID-19 pandemic on mental health as it is an ongoing global health crisis. While much of the focus has been on physical symptoms and outcomes, there is a growing recognition of the impact that COVID-19 can have on mental health. This information is paramount for designing targeted interventions aimed at promoting the mental well-being of post-COVID syndrome patients and for identifying individuals who may be at greater risk of developing mental health conditions. Furthermore, evaluating the incidence of mental disorders can furnish valuable data for public health officials in their efforts to mitigate the overall effect of the pandemic on mental health. By evaluating and addressing the incidence of mental disorders among individuals with post-COVID syndrome, healthcare professionals can work towards improving patient outcomes and ensuring that those affected receive the necessary care and support to manage their symptoms and attain complete recovery. Understanding the prevalence and risk factors associated with these conditions is important for developing effective interventions and support for individuals affected by COVID-19, both in the short and long term.

The typical duration for recovery from COVID-19 ranges from 2 to 3 weeks, contingent upon the severity of the symptoms. However, there is still a lack of agreement regarding the appropriate definitions for scenarios in which COVID-19 symptoms persist beyond the acute phase of the infection [180]. The term "Long COVID" is commonly used to describe the condition of individuals who have recuperated from COVID-19, but exhibit persistent symptoms beyond the expected recovery period [181]. Previous research is increasingly providing evidence to suggest that a considerable percentage of individuals who have survived COVID-19 are prone to a variety of physical and psychological symptoms that persist or even emerge several months after the initial infection [182, 183]. Neuropsychiatric symptoms, such as depression, anxiety, post-traumatic stress disorder (PTSD), sleep disturbances, fatigue, and cognitive deficits, have been reported commonly within the studies investigating long COVID [184].

Initially, research was centered on analyzing the psychosocial reaction to the COVID-19 pandemic and the effects of various preventive measures, including lockdowns, school closures, and travel restrictions, on the mental health outcomes of the general population. However, recent studies have expanded this scope to include not only the effects of pandemic-related circumstances but also the impact of COVID-19 as a debilitating disease [185, 186].

Several risk factors have been identified for mental disorders related to long COVID. These risk factors can be classified into several categories, such as demographic factors, clinical factors, and environmental factors. Demographic factors that have been associated with an increased risk of mental disorders in "long COVID" patients include older age, female gender, socioeconomic status, and pre-existing mental health conditions [180, 187, 188]. Clinical factors, such as the severity of the initial COVID-19 infection [189, 190], medical comorbidities [191], hospital admission, and a greater load of symptoms both during the acute phase of the infection and after discharge have also been identified as risk factors for mental disorders [192]. Environmental factors, such as the death of family members due to COVID-19, social distancing, and quarantine, may also increase the risk of mental health problems among long COVID patients [193]. The factors that have been identified as playing a role in determining the extent of mental health problems can be employed to screen individuals who have contracted COVID-19. This screening can facilitate the provision of timely interventions to those who continue to experience post-COVID symptoms, particularly with respect to mental health.

The underlying mechanisms responsible for depression, anxiety, and sleep disturbances among long COVID patients is a complex and multifaceted process that is not yet fully understood. Previous studies on the pathophysiology of these conditions suggest that the viral infection can directly impact the central nervous system, leading to the development of psychiatric symptoms. This process is probably caused by the invasion of the virus into the CNS and the subsequent neuronal damages including demyelination and neurodegeneration [194, 195]. Furthermore, the high levels of proinflammatory cytokines during COVID-19 have been shown to cause an increase in neuroinflammatory response, which can lead to a disruption in neurotransmitter activity and the development of mood disorders such as depression and anxiety [196]. Additionally, the inflammatory response to COVID-19 can lead to oxidative stress and mitochondrial dysfunction, which can further exacerbate neuropsychiatric symptoms [197]. Another explanation is that ischemia caused by microvascular dysfunction and thrombosis could lead to the formation of several small cerebral infarctions, resulting in persistent neurological impairment [198].

The prevalence of mental health disorders among long COVID survivors emphasizes the need for community-level interventions to promote mental health and well-being. it is evident that the care of COVID-19 patients extends beyond hospital discharge, and interdisciplinary collaboration is necessary for comprehensive outpatient care to ensure that long COVID patients who require mental health support have access to appropriate services. Physicians and healthcare workers should inform patients, particularly those in high-risk groups, of the potential long-term effects of COVID-19 and encourage them to seek medical care for any developing conditions. Healthcare providers and public health officials must work collaboratively to reduce the stigma associated with mental health issues and promote access to mental health resources and support groups. This approach will ensure that individuals with long COVID receive the necessary care and support to effectively manage their mental health.

The current study possesses strengths that contribute to the robustness of our findings and their potential implications for the field. We employed a comprehensive search protocol that encompassed all the relevant studies related to our outcomes, which ensured that our analysis was comprehensive and not limited to specific regions or countries. Additionally, we included a diverse sample of participants with varied demographic characteristics in our analysis, which increases the generalizability of our findings.

The results of our studies should be interpreted in the context of some limitations. The results obtained from studies showed a high level of heterogeneity. Moreover, the included studies were observational, therefore a causal relationship could not be established based on these findings. Furthermore, most of the studies did not include an unexposed control group making it challenging to conclude that mental disorders are direct consequences of the disease rather than the socioeconomic impacts of the pandemic. One of the limitations is the inconsistency in the definition of the long COVID timeframe employed in the different studies included in our analysis. Moreover, the identification and diagnosis of mental health disorders examined in our study relied on questionnaires that varied between the included studies. While we have conducted a comprehensive analysis based on the existing evidence regarding the prevalence of the three primary psychiatric manifestations in individuals grappling with long COVID, it is essential to highlight a critical aspect. Studies should take into account the hospitalization rates of patients and continue to monitor these individuals to determine whether those with more severe disease are more likely to experience long COVID [199]. It is worth noting that this aspect has often been overlooked, primarily due to the predominantly cross-sectional nature of most studies on this subject. Therefore, there is a compelling need for future longitudinal studies with adequate follow-up to delve deeper into this specific topic and provide a more comprehensive understanding of the relationship between disease severity, hospitalization, and the occurrence of long COVID. syndrome Altogether, these limitations may affect the comparability of the findings from different studies and the accuracy of our findings.

In conclusion, our results revealed higher rates of mental health conditions among long COVID patients than in the general population. This information is critical for designing interventions aimed at promoting the mental well-being of post-COVID syndrome patients, identifying high-risk individuals, and mitigating the overall effect of the pandemic on mental health. Further research should focus on identifying the specific mechanisms responsible for the development of mental health conditions among long COVID patients, as well as evaluating the effectiveness of interventions aimed at promoting mental well-being. Additionally, community-level interventions are necessary to promote mental health and well-being among long COVID survivors.

### Supplementary Information

**Additional file 1: Supplementary Table 1.** PRISMA 2020 checklist. **Supplementary Table 2.** Search strategies for online databases. **Supplementary Table 3.** Characteristics of the included studies. **Supplementary Fig 1.** Risk of bias assessment for each included study.

## Data Availability

All data has been presented in the manuscript.
