# Supplementary material for: The global prevalence of depression, anxiety, and sleep disorder among patients coping with Post COVID-19 syndrome (long COVID): a systematic review and meta-analysis
Source: BMC Psychiatry. 2024 Feb 6;24:105. doi: 10.1186/s12888-023-05481-6 (PMC10848453; doi:10.1186/s12888-023-05481-6)
Supplement: Supplementary file 1 — Additional file 1: Supplementary Table 1. PRISMA 2020 checklist. Supplementary Table 2. Search strategies for online databases. Supplementary Table 3. Characteristics of the included studies. Supplementary Fig 1. Risk of bias assessment for each included study. [file 12888_2023_5481_MOESM1_ESM.docx]

# Supplementary Material

**The global prevalence of depression, anxiety, and sleep disorder among patients coping with Post COVID-19 syndrome (long COVID): a systematic review and meta-analysis**

**Supplementary Table 1.** PRISMA 2020 checklist.

**Supplementary Table 2.** Search strategies for online databases

**Supplementary Table 3.** Characteristics of the included studies.

**Supplementary Fig 1.** Risk of bias assessment for each included study.

This supplemental material has been provided by the authors to give readers additional information about their work.

**Supplementary Table 1.** PRISMA 2020 checklist

| **Section and Topic** | **Item #** | **Checklist item** | **Reported on page #** |
| --- | --- | --- | --- |
| **TITLE** | | |  |
| Title | 1 | Identify the report as a systematic review. | 1 |
| **ABSTRACT** | | |  |
| Abstract | 2 | See the PRISMA 2020 for Abstracts checklist. | 2 |
| **INTRODUCTION** | | |  |
| Rationale | 3 | Describe the rationale for the review in the context of existing knowledge. | 3 |
| Objectives | 4 | Provide an explicit statement of the objective(s) or question(s) the review addresses. | 4 |
| **METHODS** | | |  |
| Eligibility criteria | 5 | Specify the inclusion and exclusion criteria for the review and how studies were grouped for the syntheses. | 4-5 |
| Information sources | 6 | Specify all databases, registers, websites, organisations, reference lists and other sources searched or consulted to identify studies. Specify the date when each source was last searched or consulted. | 4-5 |
| Search strategy | 7 | Present the full search strategies for all databases, registers and websites, including any filters and limits used. | Suppl. |
| Selection process | 8 | Specify the methods used to decide whether a study met the inclusion criteria of the review, including how many reviewers screened each record and each report retrieved, whether they worked independently, and if applicable, details of automation tools used in the process. | 4-5 |
| Data collection process | 9 | Specify the methods used to collect data from reports, including how many reviewers collected data from each report, whether they worked independently, any processes for obtaining or confirming data from study investigators, and if applicable, details of automation tools used in the process. | 4-5 |
| Data items | 10a | List and define all outcomes for which data were sought. Specify whether all results that were compatible with each outcome domain in each study were sought (e.g. for all measures, time points, analyses), and if not, the methods used to decide which results to collect. | 4-5 |
|  | 10b | List and define all other variables for which data were sought (e.g. participant and intervention characteristics, funding sources). Describe any assumptions made about any missing or unclear information. | 4-5 |
| Study risk of bias assessment | 11 | Specify the methods used to assess risk of bias in the included studies, including details of the tool(s) used, how many reviewers assessed each study and whether they worked independently, and if applicable, details of automation tools used in the process. | 4-5 |
| Effect measures | 12 | Specify for each outcome the effect measure(s) (e.g. risk ratio, mean difference) used in the synthesis or presentation of results. | 4-5 |
| Synthesis methods | 13a | Describe the processes used to decide which studies were eligible for each synthesis (e.g. tabulating the study intervention characteristics and comparing against the planned groups for each synthesis (item #5)). | 4-5 |
|  | 13b | Describe any methods required to prepare the data for presentation or synthesis, such as handling of missing summary statistics, or data conversions. | 4-5 |
|  | 13c | Describe any methods used to tabulate or visually display results of individual studies and syntheses. | 4-5 |
|  | 13d | Describe any methods used to synthesize results and provide a rationale for the choice(s). If meta-analysis was performed, describe the model(s), method(s) to identify the presence and extent of statistical heterogeneity, and software package(s) used. | 4-5 |
|  | 13e | Describe any methods used to explore possible causes of heterogeneity among study results (e.g. subgroup analysis, meta-regression). | 4-5 |
|  | 13f | Describe any sensitivity analyses conducted to assess robustness of the synthesized results. | 4-5 |
| Reporting bias assessment | 14 | Describe any methods used to assess risk of bias due to missing results in a synthesis (arising from reporting biases). | 4-5 |
| Certainty assessment | 15 | Describe any methods used to assess certainty (or confidence) in the body of evidence for an outcome. | 4-5 |
| **RESULTS** | | |  |
| Study selection | 16a | Describe the results of the search and selection process, from the number of records identified in the search to the number of studies included in the review, ideally using a flow diagram. | 6, Figure 1 |
|  | 16b | Cite studies that might appear to meet the inclusion criteria, but which were excluded, and explain why they were excluded. | Figure 1 |
| Study characteristics | 17 | Cite each included study and present its characteristics. | 6, suppl. |
| Risk of bias in studies | 18 | Present assessments of risk of bias for each included study. | suppl. |
| Results of individual studies | 19 | For all outcomes, present, for each study: (a) summary statistics for each group (where appropriate) and (b) an effect estimate and its precision (e.g. confidence/credible interval), ideally using structured tables or plots. | Figures, suppl. |
| Results of syntheses | 20a | For each synthesis, briefly summarise the characteristics and risk of bias among contributing studies. | 6-7 |
|  | 20b | Present results of all statistical syntheses conducted. If meta-analysis was done, present for each the summary estimate and its precision (e.g. confidence/credible interval) and measures of statistical heterogeneity. If comparing groups, describe the direction of the effect. | 7-9 |
|  | 20c | Present results of all investigations of possible causes of heterogeneity among study results. | Suppl. |
|  | 20d | Present results of all sensitivity analyses conducted to assess the robustness of the synthesized results. | 6-7 |
| Reporting biases | 21 | Present assessments of risk of bias due to missing results (arising from reporting biases) for each synthesis assessed. | 6-7 |
| Certainty of evidence | 22 | Present assessments of certainty (or confidence) in the body of evidence for each outcome assessed. | 6-7, suppl. |
| **DISCUSSION** | | |  |
| Discussion | 23a | Provide a general interpretation of the results in the context of other evidence. | 7 |
|  | 23b | Discuss any limitations of the evidence included in the review. | 7-10 |
|  | 23c | Discuss any limitations of the review processes used. | 7-10 |
|  | 23d | Discuss implications of the results for practice, policy, and future research. | 7-10 |

**Supplementary Table 2.** Search strategies for online databases

| **MEDLINE (via PubMed)** | 1- “Post-Acute COVID-19 Syndrome” [mh] OR Post-Acute COVID-19 Syndrome[tiab] OR Post-Acute COVID-19 Syndromes[tiab] OR Long Haul COVID-19[tiab] OR Post Acute COVID-19 Syndrome[tiab] OR Long COVID[tiab] OR Post-Acute Sequelae of SARS-CoV-2[tiab] OR Long-Haul COVID[tiab] OR Post-COVID[tiab] OR persistent covid[tiab] OR COVID-19 sequelae[tiab] OR long-tail covid[tiab] OR COVID-19 survivors[tiab] OR SARS-CoV-2 survivors[tiab]  2- - “Depressive Disorder”[mh] OR “Depression”[mh] OR “Anxiety”[mh] OR “Sleep Wake Disorders”[mh] OR “Depression “[tiab] OR “Depressive “[tiab] OR “Depress*”[ tiab] OR “Anxiety”[ tiab] OR “Anxieties “[tiab] OR “Anxiousness”[ tiab] OR “Sleep”[ tiab]  3- “Prevalence” [mh] OR “Epidemiology” [mh] OR Prevalence[tiab] OR Epidemiolog*[tiab] |
| --- | --- |
| **Embase** | 1- ‘long COVID’/exp OR ‘Post-Acute COVID-19 Syndrome’:ti,ab OR ‘Post-Acute COVID-19 Syndromes’:ti,ab OR ‘Long Haul COVID-19’:ti,ab OR ‘Post Acute COVID-19 Syndrome’:ti,ab OR ‘Long COVID’:ti,ab OR ‘Post-Acute Sequelae of SARS-CoV-2’:ti,ab OR ‘Long-Haul COVID’:ti,ab OR ‘Post-COVID’:ti,ab OR ‘persistent covid’:ti,ab OR ‘COVID-19 sequelae’:ti,ab OR ‘long-tail covid’:ti,ab OR ‘COVID-19 survivors’:ti,ab OR ‘SARS-CoV-2 survivors’:ti,ab  2- ‘Depression’/exp OR ‘Anxiety’/exp OR ‘Depression ‘:ti,ab OR ‘Depressive ‘:ti,ab OR ‘Depress*’:ti,ab OR ‘Anxiety’:ti,ab OR ‘Anxieties ‘:ti,ab OR ‘Anxiousness’:ti,ab OR ‘Sleep’:ti,ab  3- ‘prevalence’/exp OR ‘epidemiology’/exp OR ‘prevalence’:ti,ab OR ‘epidemiolog*’:ti,ab |
| **Web of Sciences/Scopus** | 1-“Post-Acute COVID-19 Syndrome” OR “Post-Acute COVID-19 Syndromes” OR “Long Haul COVID-19” OR “Post Acute COVID-19 Syndrome” OR “Long COVID” OR “Post-Acute Sequelae of SARS-CoV-2” OR “Long-Haul COVID” OR “Post-COVID” OR “persistent covid” OR “COVID-19 sequelae” OR “long-tail covid” OR “COVID-19 survivors” OR “SARS-CoV-2 survivors”  2- “Depression “OR “Depressive “OR “Depress*”OR “Anxiety”OR “Anxieties “OR “Anxiousness”OR “Sleep”  3-“prevalence” OR “epidemiolog*” |

**Supplementary Table 3.** Characteristics of the included studies.

| **Author** | **Year** | **Study design** | **Total Patients (n)** | **Age (mean ± SD)** | **Male (%)** | **Assessment scales** | **Rate of response** | **Cut-off value** | **Hospitalization rate at baseline%** | **Severe infection %** | **Follow up duration** |
| --- | --- | --- | --- | --- | --- | --- | --- | --- | --- | --- | --- |
| Albu | 2021 | single center cross-sectional observational study | 30 | mean : 54 | 63.33 | The Pittsburgh Sleep Quality Index HADS | 96.66% | HADS : 8–10 Mild  11–14 Moderate  15–21 Severe The Pittsburgh Sleep Quality Index : ranging from 0 to 21 0–4 indicating “good” sleep and 5–21 indicating “poor” sleep | NA | NA | 18.5 months |
| Abramoff | 2023 | cohort | 324 | 46.6 (14.0) | 31.2 | HADS | NR | scored >8 | 23 | 4.37% | NA time since covid dx : 0 - plus 30w) |
| Agergaard | 2022 | cohort | 547 | 47 (IQR 36–56) | 28 | Depression: SCL-13.Anxiety: SCL-13 | 82% | score > 8,score > 5 | 12 | NA | median of 6.3 (IQR 4.4–9.9) months after the onset of symptoms |
| Ahmed | 2021 | cross-sectional study | 182 | 46.49 ±17.4 | 46.20% | 1.Pittsburgh sleep quality index "PSQI” 2.Symptom checklist 90 "SCL 90 | | 1:05 | 100 | 62.30% | six months |
| Ajmi | 2022 | cross-sectional study | 50 | 19-86 | 62% | Hospital Anxiety and Depression Scales (HADS) | NR | NR | 12 | 12% | 1 to 3 months after a diagnosis of COVID-19 |
| Akçay | 2022 | cross-sectional | 74 | adolescents : 14.8 (±1.8) parents :NA | adolescents :44.59 parents : 25.7 | RCADS UCLA-LS DASS-21 | NR | RCADS :65–69 (borderline clinical), ≥70 (clinical UCLA-LS: 20-80 DASS-21:(>9 points for depression; >7 points for anxiety; > 14 points for stress) | NA | NA | NA |
| Albtoosh | 2022 | cohort | corrected : 472 COVID-19 survivors = 125 control = 347 | NR | 53.10% | anxiety : GAD-7/depression : PHQ-9. | 64.7٪ | NR | 60NA8 | NA | 43.2% infected > 12 months before the interview 28.8% were infected < 6 months  28.0% were infecteds 6–12 months |
| Ali | 2021 | cross-sectional study | 1108 | 18 years or  older most participants were in the age groups 18–30 years (56.1%) and 31–40 years (30.4%) | 59.80% | (DASS-21) | NR | NR | NA | NA | NA |
| Anik | 2023 | cross-sectional | 481 | 45.3 (±14.2) | 60.3 | DASS-21 | 51% | DASS-D≤9.DASS-A | 100 | NA | on average 6 months after their discharge |
| Azevedo | 2022 | Prospective cohort | 389 | 51.82 (14.04) | 38.30% | Hospital Anxiety and Depression (HAD) scale | NR | HADS ≥ 8 | 61 | 52% | NA |
| Badinlou | 2022 | a web-survey | 507 | 47.72 ±10.62 | 12 | PHQ-9 GAD-7 ISI-7 | 100 | PHQ-9: 0-27 GAD-7 : 0-21 ISI-7: 0-28  CUTOFF:≥10 | NA | NA | NA |
| Bahadur | 2022 | cross-sectional | 42 | 28.7 ± 5.36 | NA | DASS-21 | NR | NR | 100 | *Women requiring intensive care unit admission with severe symptoms were excluded from the study | 3 months |
| Bai | 2022 | a prospective cohort | corrected: 377 | 57 (IQR 49-68) | 64 | HADS | 77% | HADS>8 | 100 | NA | 102 (IQR 86–126) days from acute symptom onset |
| Boesl | 2021 | cohort | 100 | mean:45.8 | 33% | (Beck Depression Inventory Version I, BDI) | NR | >10 | 11 | 11% | mean, 184.5 days |
| Boiko | 2022 | cohort retrospective | 278 | 39.6 ± 3.8 | 54% | STAI | NR | It is considered that < 30 points are a low level of anxiety, 31–45 points—moderate, > 45 points—high | NA | NA | 7.6 ± 1.1 weeks |
| Bonazza | 2022 | cohort | 100 | 58.7 ± 11.8 | 72 | HADS | 71.9% | ≥ 11 | 100 | NA (CPAP = 31% , ICU admit = 7%) | 6 months |
| Braga | 2022 | cross-sectional | 614 | 47.6± 11.2 | 27% | HADS | NR | 11 | 33 | 33% | an average of eight months |
| brown | 2022 | observational cohort | 297 | 46.9± 14.1 | 29.90% | HADS | NR | >8 | 23NA6 | NA (ICU admit = 6.7%) | NA |
| Bungenberg | 2022 | crosssectional | 50 | 50.5 | 44% | HADS | NR | ≥10 | 42 | NA | *Median time from SARS‐CoV‐2 detection to investigation was 29.3 weeks |
| Cacciatore | 2022 | secondary analysis of cohort | 105 | 66.9 | 75.90% | PSQI and HADS | 79% | HADS—anxiety and HADS—depression≥ 8 and sleep disturbances PSQI ≥5 | NA | NA | variable in consideration of patients |
| Cacciatore | 2022 | a secondary analysis of the NEXT study | 83 | MEDIAN : 66.9 | 75.9 | HADS PSQI | NR | HADS score ≥ 8 : relevant anxiety or depression PSQI score ≥ 5 : poor sleep | 100 | NA | variable in consideration of patients’ clinical status and pandemic situation |
| Campo-Arias | 2021 | cross sectional | 330 | 47.67 ±15.17 | 38.40% | Athens Insomnia Scale (AIS) and PHQ9 | NR | PHQ9 ≥7 and AIS ≥6 | NA | NA | NA |
| Cansei | 2021 | cross-sectional study | 102 | 44.4±13.7 | 55.90% | PHQ9 and GAD7 | NR | GAD7 ≥15 and PHQ9 ≥20 | NA | NA | NA |
| Casoni | 2022 | prospective cohort | 57 | 66.42±12.71 | 59.6% | Sleep diaries and validated questionnaires | NR | 9.28 | 100 | NA | 3 months |
| Chen | 2022 | prospective, closed cohort study | 200 | 44.6 | 35.50% | PHQ-9, GAD-7 | NR | ≥ 10 for depression and ≥ 8 for anxiety | NA | NA | 125 days |
| Cheung | 2023 | prospective cohort study | 475 | 46.7 | 30% | GAD-7 | 30% | ≥ 10 | NA | NA | 306 days |
| Choudhry | 2021 | cohort | 445 | 42±10 | 65.3% | PSQI | NR | 5 | NA | NA | 30 days |
| Clemente | 2022 | observational cohort | 93 | Control=60.62 ± 17.37/Long-COVID:62.54 ± 10.18 | 70.83% | Symptom Checklist-90 (SCL90) | NR | NR | 100 | NA | three months after (95 ± 10 days) their discharge from the COVID |
| Clift | 2022 | cohort | 8380000 | 49.07 (18.40) | 50 | ICD-10 | NR | NR | 0NA6 | 0.02% | 1 Year |
| Cook | 2022 | retrospective cohort study | 472 | 46 | 48% | PHQ-2, GAD-2 | 100% | NA | NA | NA | NA |
| Dai | 2021 | cohort | 50 | 48 ± 14 | 50 | DASS-21 | 80% | moderate depression = 14-20 - moderate anxiety = 10-14 | 100 | 22% | 6 months |
| Dale | 2022 | cross-sectional study | 98 | 46.8 | 39% | PHQ-2 | NR | ≥ 3 | NA | NA | NA |
| Damiano | 2022 | cohort study | 425 | 55.7, SD 14.2 | 51.53 | HADS | NR | NR | NA | NA | 6 months |
| Dankowski | 2021 | cross‑sectional observational | 102 | 52±13 | 45% | BDI and STAI | NR | bdi≥12 and stai≥10 | 23NA5 | 23.6% | 56 ±18 days |
| Dar | 2021 | cross-sectional study | 127 | 54.2 ± 4.22 | 65.50% | HADS | 93% | ≥ 8 | NA | NA | NA |
| Diem | 2022 | survey study | 402 | 44.6 ± 1.4 | 19.4 | FSS, ESS, ISI, and WHO‐5 Well‐being Index | 76.86% | NR | 10NA7 | NA | 13 months |
| Diem | 2022 | survey study | 42 | 44.8 | 45.20% | BDI II, ESS | NR | NR | NA | 19.10% | 28 weeks |
| Dondaine | 2022 | cross-sectional | 62 | O2 posetive: 52.19 ±8.72 O2 negative : 43.90 ±10.92 | 41.93 | CES-D HAM-A ESS | NR | CES-D : women ≥23 men≥17 HAM-A≥20 ESS≥10 | NA | NA | 3–9 months |
| Elmazny | 2023 | cross-sectional | 1638 | 38.28±13.004 | 34.73 | DSM-5 | NR | NR | NA | 4.80% | 3 months |
| Eloy | 2021 | prospective cohort | 324 | 61 | 63% | HADS | NR | ≥11 | NA | 10% | six-months |
| Ettman | 2020 | survey study | 1441 | 18 -39 | 50.20% | PHQ9 | NR | ≥10 | NA | NA | telephone follow up |
| Ezzelregal | 2021 | cross-sectional study | 102 | 35–55 years | 39.20% | Beck depression inventory score | NR | NR | 0 | 0 | NA |
| Fernández-de-las-Peñas | 2021 | cross-sectional | 1200 | 61 ± 17 | 52 | HADS-A, HADS-D, and PSQI | 95.16% | HADS-A ≥ 12 points, HADS-D ≥ 10 points, PSQI> 8.0 | 100 | NA (ICU admit = 6.6%) | Participants were assessed a mean of 8.4 months (SD, 1.5) |
| Fernández-de-Las-Peñas | 2021 | case-control study | 264 | 52.0 ± 14.5 | 60.20% | HADS, PSQI | 100% | HADS-A ≥ 12; HADS-D ≥ 10; PSQI ≥ 8 | NA | NA | 7.2 months |
| Fernández-De-las-peñas | 2022 | Multicenter cohort Study | 1969 | age: 61,  SD: 16 years | 53.70% | Hospital Anxiety and Depression Scale (HADS) and Pittsburgh Sleep Quality Index (PSQI) | NR | anxiety: (HADSA ≥12 points)  depressive symptoms :(HADS-D ≥10 points) PSQI:scores ≥8.0 points suggest poor sleep quality | 100 | 6.60% | mean of 8.4 months |
| Fernández-de-las-Peñas | 2022 | case–control study | 574 | Hypertensive:68.3 (12.6) Normotensive:68.3 (12.5) | Hypertensive:(39.7% Normotensive:39.7% | The Hospital Anxiety and Depression Scale (HADS) and the Pittsburgh Sleep Quality Index (PSQI) were used to assess anxiety/ depression symptoms and sleep quality | | (HADS-A ≥ 12 points; HADS-D ≥ 10 points)  for determining anxiety and depressive symptoms score ≥8.0 points suggests poor sleep quality | 100 | 14.20% | mean of 7.2 months |
| Fernández-de-las-Peñas | 2022 | cohort | 201 | 56.5±21 | 46.6 | HADS/Pittsburgh Sleep Quality Index (PSQI | 100% | HADS-A≥12 /HADS-D≥10.PSQI ≥8 points | 100 | NA | 6 months |
| Ferrando | 2022 | cross-sectional study | 60 | 41.4 ± 13.5 | 32% | PHQ-9, GAD-7 | NR | PHQ-9 ≥ 11, GAD-7 ≥ 10 | NA | NA | 6 months |
| Fischer | 2022 | cohort | 288 | 43 ± 13 | 41% | PSQI | NR | >5 | NA | 14.93% | 12 months |
| Frontera | 2022 | prospective, longitudinal cohort study | corrrected: 790 | 6-month: 69 and 12-month: 65 | 6-month: 65% and 12-month: 64% | NeuroQoL | 6-month: 48% and 12-month: 41% | ≥ 60 | 100 | NA | 12months |
| Fu | 2022 | case-control study | 267 | N/A | 46.70% | SQS, PSQI, MQI | 74.50% | 0 = terrible, 1-3 = poor,4-6 = fair,7-9 = good, and 10 = excellent | NA | 12.1 | 6 months |
| Fu | 2021 | cross-sectional | 199 | NA | 46.70% | single-item Sleep Quality Scale; SQS | The response rate  was 74.5% | Cut-off scores of 1, 4, 7, and 10 were used to define poor, fair, good and excellent sleep quality | 100 | 12.10% | 6 months |
| ghosn | 2023 | prospective cohort | 737 | 61 | 64% | HADS | 43% | >10 | 100 | NA | 12 months |
| Gorelik | 2023 | cohort | 2295 | 45.6 ± 14 | 43% | Well-Being Index (WHO-5) | NR | 11.8 | NA | NA | NA |
| Gramaglia | 2022 | prospective cohort study | 200 | 61.5 | 61% | BDI-II, BAI | 97.50% | BDI-II > 13, BAI > 21 | NA | NA | 4 to 12 months |
| Grover | 2021 | cross-sectional study | 206 | 36.08 ± 13.12 | 53.90% | PHQ-4 | NR | ≥ 3 | NA | NA | NA |
| Guido | 2022 | cohort study | 322 | 9.53 ± 3.73 | 51.86% | MASC 2-SR, CDI-2 SR | NR | > 65 | NA | NA | 5 monts |
| Guo | 2023 | prospective cohort study | 208 | 58 | 48.10% | PHQ-9, GAD-7 | NR | ≥ 5 | NA | 29.80% | 18.5 months |
| Haag | 2022 | longitudinal study | 233 | 19.6 ± 6 | 45.10% | PHQ-9, GAD-7 | NR | N/A | NA | NA | 6 months |
| Hammerle | 2023 | cross-sectional | 127 | Median : 42 | 23.6 | HADS | NR | below 7 : improbable anxiety/depression,   8 to 11 : probability of anxiety/depression, and scores  12 and 21 : possible anxiety/depression. | 7NA1 | 0% | median of 7 months |
| Harrison | 2022 | observational cross-sectional study | 300 | 58.11 | 56% | HADS-A | NR | ≥ 11 | NA | NA | telephone follow up |
| Hartung | 2022 | cohort | 969 | NA | 45 | Depressive:PHQ-8 .anxiety:GAD-7.leep disturbances :PSQI | NR | PSQI ≥5 | NA | 1% | NA (interquartile range 8 to 12 months)Median = 9 months after SARS-CoV-2 infection |
| Hayek | 2021 | cross-sectional study | 52 | 57.33 | 46.20% | We retrieved and collected data from patients’ Electronic Health Records available on the EPIC Systems at AUBMC | NR | NR | NA | NA | NA |
| Hazumi | 2022 | cross-sectional | 6016 | 30_49 | 42.47% | Patient Health Questionnaire-9 and | NR | ≥ 10 | NA | NA | ≥6 months |
| Heesakkers | 2022 | a multicentre prospective  cohort study | 166 | 57.8 + 11.4 | 21.10% | HADS | 166/197(84.3) | HADS ≥ 8 | 100 | 100% | 3 and 12 months after ICU admission |
| Henneghan | 2021 | observational, cross-sectional study | 57 | 37.33 | 21.15 | PROMIS | 91.22% | NR | NA | 5.77% | NA |
| Houben-Wilke | 2022 | follow up study | 239 | 50 | 17.20% | HADS | 100% | ≥ 8 | NA | NA | 6 months |
| Huang | 2022 | cross-sectional | 574 | 57.67 ± 11.42 | 39.4 | GAD-7, *a scale for sleep disorders wasn’t defined | 25.76 | GAD-7 ≥ 10 | 100 | 19.7% | 6 months |
| Huang | 2022 | cohort study | 511 | 56.23 ± 12.18 | 51.86% | PHQ-9, GAD-7 | NR | ≥ 9 | NA | 19.70% | 193.9 days |
| Huarcaya-Victoria | 2023 | single-center cross-sectional study | 318 | 53.1 | 61.30% | PHQ-9 and GAD 7 | NR | The PHQ-9 scores reflect 5 categories of severity of depressive disorders:None (0–4), mild (5–9), moderate (10–14), moderately severe (15–19), and severe (20–27) .GAD-7 reflects 4 categories of severity of the anxiety disorder: none (0–4), mild (5–9), moderate (10–14),and severe anxiety (15–21). | NA | NA | NA |
| Hüfner | 2022 | cohort study | 1157 | 43 | 35% | PHQ-4 | NR | ≥ 3 | NA | NA | 79 and 96 days in the Austria and Italy |
| Imran | 2021 | prospective multicentric cross-sectional study | 104 | 40 | 67% | PHQ-9, GAD-7 | 30 DAYS: 99%, 60 DAYS: 81.7% | PHQ-9 ≥ 10, GAD-7 ≥8 | NA | 9.70% | 60 days |
| Ingibjörg Magnúsdóttir | 2022 | follow-up study | 247249 | 46.6(sd:12.9) | 32.1 | PHQ-9 EST-Q2 PSQI DSM-5 GAD-7 ASS | NR | Depression subscale cutoff of >11 General Anxiety Disorder ≥10 Anxiety subscale >11 PTSD Screen for DSM-5 ≥4 PHQ - binary with cutoff >= 10 * GAD - binary with cutoff >= 10 * Sleep length - binary with cutoff < 6 hours * Sleep quality - binary with cutoff "Fairly bad"/"Very bad" vs. "Fairly good"/"Very good Insomnia subscale >5 | NA | NA | NA |
| Islam | 2021 | cross sectional | 1002 | 34.7 ±13.9 | 57.90% | PHQ9 | NR | PHQ9 ≥ 20 | NA | NA | NA |
| jafri | 2022 | cross sectional | 70 | 26.29 + 11.79 | 40% | patient health questionnaire-9(PHQ-9) and corona anxiety scale (CAS). | NR | NR | NA | NA | NA |
| jung | 2022 | retrospective cohort | 1122 | 51.4 ± 15.3 | 34% | HADS | NR | >7 | NA | NA | NA |
| Kalamara | 2022 | cohort | 133 | 56.0 ± 11.48 | 59.4% | PSQI | NR | ≥ 5 | NA | 31.20% | 6 months |
| Kamran | 2022 | cross-sectional | 324 | NA | 31% | GAD-7 | 44.7 | Scores of 5, 10,and 15 were considered as the cut-off points for mild, moderate and severe anxiety, respectively | NA | NA | 6 months |
| Kaur | 2021 | prospective cohort study | 440 | 44.23 ± 12.56 | 59.10% | PHQ‑9 | NR | NR | NA | NA | NA |
| Kaur | 2023 | cohort study | 160 | 38.8 ± 10.9 | 66.25% | ICD-10 | NR | NR | NA | NA | NA |
| Khatun | 2023 | cross-sectional study | 325 | 44.34 6 13.87 | 61.50% | PHQ-9 | NR | NR | NA | NA | NA |
| Korkut | 2022 | cross-sectional study | 339 | 38.33 ± 11.12 | 45.10% | GAD-7, CAS | NR | GAD-7 ≥ 8, CAS ≥ 5 | NA | NA | NA |
| Larsson | 2022 | cross-sectional study | 168 | 64 | 69% | HADS | 168/211 | cutoff >7 | 100 | 53% | 3 months after hospital discharge |
| Li | 2022 | cross-sectional study | 535 | 50.80 ± 14.44 | 216 (40.4%) |  | 94.50% | NR | 100 | 17.20% | 1 year |
| Lier | 2022 | cohort study | 219 | median:49 | 35.50% | Patient health questionnaire-9 (PHQ-9) Generalized anxiety disorder scale 7 (GAD-7) | NR | phq9:10 GAD7:10 | 1 | 0.95% | median time of consultation was 6 months post infection |
| Liyanage-Don | 2021 | cross-sectional | 144 | 56± 16 | 59.70% | PHQ 8 | NR | ≥ 10 | NA | NA | 12 months |
| Magdy | 2022 | case-control | 408 | Migraine Patients : median=30 Controls :median = 36 | 23 | dsm-5 | NR | NR | 100 | 2% | 3-months after the acute phase of COVID-19 |
| Maley | 2022 | cohort | 60 | 59 ± 13 | 48.3 | HADS-A, HADS-D | 95.23% | HADS-A and HADS-D ≥ 11 points | 100 | *all particioients had undergone mechanical ventilation | 6 months |
| Mantovani | 2022 | cohort | 37 | 51.9 ± 10.9 | 67.50% | PSQI  HADS | 37/131 | PSQI:6 HADS anxiety:5 HADS depression:1 ? | 89 | 21% | > 6-month |
| Marasco | 2022 | prospective, multicentre, controlled study | 614 | 49.9±16.1 | 59.90% | HADS | 614/2183 | HADS: 8–10= borderline abnormal; 11–21= abnormal | 100 | NA | after 1, 6 and 12 months post hospitalisation |
| Matsumoto | 2022 | cross-sectional | 763 | 36.7 ± 15.1 | 63.8 | GAD-7/Patient Health Questionnaire (PHQ-9) | NR | PHQ ≥ 10/GAD ≥ 10 | 50 | NA | NA |
| Mazhari | 2022 | cross-sectional | 200 | 45.4 ±13 | 35 | HADS A-D.HAM A-D | 30%- 60/200 | NR | 100 | NA | NA |
| Mazza | 2021 | prospective cohort study | 226 | 58.52(12.79) | 65.9 | DSM-5 ZSDS BDI-13 clinical interview STAI-Y WHIIRS | NR | ZSDS index ≥ 50; BDI-13 ≥ 9; STAI-state ≥ 40; WHIIRS ≥ 9 | 52 | NA | 3 months |
| Mazza | 2022 | follow-up study | 495 | 57.6 ± 11.5 | 65.85 | BDI-13 ZSDS | NR | BDI sum score ≥ 9 ZSDS index ≥ 50 | 100 | NA | one, three, six, and twelve-month |
| Mazza | 2022 | cohort | 216 | 60.13 ± 12.21 | 69 | Depression:ZSDS.STAI-Y: Anxiety | NR | ZSDS index ≥50 | NA | NA | 12months |
| Mei | 2021 | follow-up study | 4328 | median : 59 | 45.9 | GAD-7 PHQ-9 | NR | NR | 100 | NA | median duration of the follow‐up period was 144.0 days |
| Mendes Paranhos | 2022 | cross-sectional | 219 | 43.2 ± 12.9 | 25.1 | *not reported | 87.6% | NR | 15 | NA | NA |
| Méndez | 2021 | observational longitudinal study | 171 | median : 58 | 57.9 | A battery of standardised instruments validated in the Spanish general population | NR | NR | NA | NA | NA |
| Méndez | 2021 | cross-sectional prospective study | 179 | MEDIAN : 57 | 58.7 | PHQ-2 GAD-7 | NR | NR | 100 | NA | 2 months after discharge |
| Menges | 2021 | population-based cohort study | 431 | median:47 | 217 (50.3%) | All data was collected through the Research Electronic Data Capture (REDCap) survey system. depression :(DASS-21) | 431/4639 | NR | 18 | 38.10% | median of 7.2 months (range 5.9 to 10.3 months) after their diagnosis |
| Moura | 2022 | a prospective cohort study | 189 | 45.8 | 34.90% | Geriatric Depression Scale (GDS) Beck Inventory | 189/207 | cutof point of 3 on the GDS and 10 on the Beck inventory for the diagnosis of depression | NA | NA | NA |
| Moy | 2022 | cross-sectional | 732 | 40.2±10.9 | 41.3 | PHQ-9 | 77.50% | NR | 23 | 11.70% | between infection and data collection : 27.3 ± 12.5 weeks |
| Müller | 2023 | cohort | 127 | 50.62 ±10.74 | 23.6 | HADS-D HADS-A | NR | between 8 and 10 indicated mild symptoms above 14 indicated moderate to severe symptoms | 26 | 36/127 | variable |
| Murga | 2021 | case-control | 84 | case = 44.41 ± 9.37 - control = 45 ± 13.5 | case: 21.42 - control: 36.36 | HADS-A, HADS-D, and Pittsburgh | 93.8% | NR | NA | NA | NA |
| Naidu | 2021 | cohort | 760 | 57.7 | 60.20% | Patient Health Questionnaire 2-item (PHQ-2) | 80% | NR | NA | NA | NA |
| Naik | 2022 | cross-sectional | 1094 | Hospitalized=57.3 ± 14.3 Non-hospitalized=45.4 ± 12.9 | Hospitalized=58.8 Non-hospitalized=32.1 | EQ-5D-5L PHQ-2 | NR | EQ-5D-5L: no problems, slight problems, moderate problems, severe problems or extreme problems  PHQ-2 core ≥ 3 is considered a positive screen, | 39 | 193/548 hospitalized | Hospitalized patients answered questionnaires at a mean of 111.0 days since diagnosis, were a mean age of 57.3, 44.2% were female and 31.6% were white. Non-hospitalized patients answered questionnaires at mean of 158.6 days since their COVID-19 diagnosis |
| Nehme | 2022 | cohort | 3515 | 45.2 ± 14.5 | 38.8 | HADs/Insomnia Severity Index | 46.60% | NR | 0 | NA | 12months |
| Niedziela | 2022 | cohort study | 200 | Nonhospitalized:49.0 (41.0–56.0) Hospitalized:59.0 (50.0–59.0) | 50.50% | (Hospital Anxiety and Depression Scale Athens Insomnia Scale | NR | NR | NA | NA | NA |
| Noviello | 2021 | controlled cohort | 164 | 44.1 | case: 59.8 | HADS-A, HADS-D, and SAGIS | NR | HADS-A and HADS-D ≥ 11 points | NA | 13% | 4.8 ± 0.3 months |
| Ocsovszky | 2022 | non-hospitalized cohort | 166 | 39 ± 14 | 43.4 | BDI BAI | NR | BDI ≥10 BAI : NA | 0 | NA | mean follow-up time was 25 ± 18 weeks after the diagnosis |
| Pappa | 2022 | cross-sectional | 143 | 57.1 ± 11 | 63.64 | HADS, AIS, and EQ-5D-5L | 93% | HADS (mild:8-10 , moderate: 11-14, severe: 15-21) - AIS >6 | 100 | severe: 48.25 , critical: 13.99 | 2 months |
| Park | 2021 | cohort | 260 883 | NA | 53 | ICD-10(F40–48) | NR | NR | NA | NA | 11 months |
| Pasin | 2023 | cross-sectional | 80 | 42.57 ± 11.71 | 16.3 | BAI | NR | BAI: 0 to 7 minimal anxiety, 8 to 15 points mild, 16 to 25 points moderate, and 26 to 63 points severe anxiety | 0 | NA | NA |
| Patel | 2022 | cohort study | 123757 | 61 ± 15.4 | 89% | VA’s Corporate Data Warehouse (CDW) | 123757/778,738 | NR | NA | NA | NA |
| Pathak | 2023 | cross-sectional study | 96 | N/A | 72.91% | HAM-A, HAM-D | NR | NR | NA | 17.70% | 1 month |
| Pellitteri | 2022 | Prospective cohort | 44 | median:60 | 83% | Pittsburgh Sleep Quality Index (PSQI) , Epworth Sleepiness Scale (ESS) , Beck Depression Inventory - Short Form (BDI-SF) ,Hamilton Anxiety Rating Scale (HAS), Impact of Event Scale-Revised (IES-R), Short Form Health Survey 36 (SF36) | 44/47 | PSQI total score:Poor sleepers (> 5), ESS total score:Excessive (> 10) | NA | NA | NA |
| Peluso | 2021 | cohort | 179 | MEAN:48 | 55 | GAD-7 PHQ-8 | NR | GAD-7 : 0-21 PHQ-8 : 5-24 | 24 | 44/179 | 4-month |
| Perrot | 2022 | cohort | 178 | 59.6±11.6 | 58.90% | HADS | NR | NR | 86 | icu admission: 51.1% | NA |
| Pistarini | 2021 | cross-sectional and exploratory study | 40 | 64.13 ± 11.85 | 63% | Hamilton Depression Rating Scale | NR | Hamilton Depression Rating Scale (>8) | NA |  | NA |
| Pudlo | 2022 | cross-sectional | 200 | not reported | 50.5 | The Athens Insomnia Scale (AIS) | NR | AIS ≥6 (sleep disturbences) and ≥ 11 (insomnia) | 43NA5 | NA | 1 month |
| Rass | 2022 | cohort | 81 | 54 | 59% | HADS A-D. | NR | mild :>7 .clinically:>10 . | 60NA8 | icu care : 25% | 12 months |
| Richter | 2022 | cross-sectional | 70 | MEDIAN:50.5 | 31.4 | HADS | NR | HADS depression:  ≥ 8 HADS anxiety ≥ 9 | 17 | 10/70. | at least 12 weeks ago |
| Richter | 2022 | cross-sectional study | 70 | median age: 50.5 | 31.40% | The Hospital Anxiety and Depression Scale (HADS) | 70/84 | HADS depression≥8 HADS anxiety≥8 | NA | NA | NA |
| Rousseau | 2021 | single-center cohort study | 32 | M3 Cohort=62 | 72 | PSQI HADS-A HADS-D | NR | HADS> 7 PSQI ≥ 5 | 100 | 32/32 | 94 [90–101] days after ICU discharge |
| Salehian | 2022 | cross-sectional | 158 | 42.02 ± 10.56 | 58.2 | HADS | 39.5% | HADS: 8 - 10 as borderline and 11 or more as significant | 100 | NA | 1 month |
| Salimi | 2021 | cross-sectional | 188 | 56.4 ± 9.6 | 62.2 | HADS and Anxiety and Stress Scale 42 (DASS-42) | NR | HADS ≥ 11 points , cut-off for DASS-42 was not reported | 100 | NA | NA |
| Santos | 2022 | cohort | 61 | 29.85 ± SD not reported | NA | Edinburgh Postnatal Depression Scale (EPDS) | case: 47.6% - control: 69.3% | EPDS ≥ 11 | NA | NA (among mild ones) | average = 6 months after the infection |
| Sathyamurthy | 2021 | cohort | 279 | 71 ±5.56 | 63.80% | Geriatric Depression Scale-5 . Geriatric Anxiety | | NR | 100 | NA | (After 90 days of recovery) |
| Sayde | 2023 | cohort | 21 | 68 ± SD not reported | 95.2 | PHQ-9 and GAD-7 | 47.72 | moderate: PHQ-9 and GAD-7 >10 - severe: PHQ-9 and GAD-7 >15 | 100 | * all patients were post-ICU survivers | 6 months |
| Schneider | 2023 | cross-sectional | 2828 | 47.3 ± 17.1 | 46.20% | PHQ-2 (depression), GAD-2 (anxiety) | 31.70% | PHQ-2,GAD-2 ≥3 | NA | NA | (Months since infection =9.2 ± 6.0) |
| Schulz | 2022 | case-control study | 160 663 | median:51 | 41.30% | HADS-A | NR | NR | NA | NA | NA |
| Schulze | 2022 | cross-sectional | 101 | 50.2± 12.9 | 26.70% | HADS/neurology consultant | NR | normal=≤7 . Pathological=>10 | 50 | icu = 4% | NA (infection was at least 2 months before) Range: 60–554 days averaged 220.1 days (SD: 118.26) |
| Senara | 2023 | case-control | 200 | case: 35.9 ± 8.5 - control: 38.1 ± 10.6 | case: 22.5 - control: 24 | Hamilton Anxiety rating scale (HAM-A) | NR | NR | NA | 24% | NA |
| Shah | 2022 | cross-sectional | 261 | males: 48.5 ± 15.7 - females: 49.2 ± 14.3 | 67.8 | PHQ-9 | 95% | PHQ-9 ≥10 | NA | * study had excluded severely ill patients who remained hospitalized for 21 days after RT-PCR testing. | immediate FU: 14-21 days short-term FU: 90-97 days |
| Shukla | 2023 | multicenter cross-sectional | 679 | 31.49 ± 9.54 | 49.19% | Depression, Anxiety, and Stress Scale-21 (DASS21) questionnaire and the modified sleep disorders questionnaire | NR | NR | 45 | severe : 0.59% | NA (between 12 and 52 weeks post discharge after COVID-19) |
| Singh | 2021 | cross-sectional | 100 | 22±9.5 | 38 | PHQ-9 GAD-7 | NR | GAD-7 : 0-21 PHQ-8 : 0-27 | NA | NA | NA |
| Speichert | 2022 | cross-sectional | 371 | not reported | 45.8 | GAD-7 and PHQ-8 | GAD-7: 88.79% - PHQ-9: 84.6% | PHQ-9 ≥5, ≥10, ≥15, and ≥20 =mild, moderate, intermediate, and severe depression - GAD-7 ≥5, ≥10, and ≥15 = mild, moderate, and severe anxiety symptoms | 36NA7 | NA | 300 days |
| Staudt | 2022 | cohort | 101 | 60 | 58% | PHQ-9-D for depression | NR | PHQ-9-D ≥ 10 | 100 | icu = 20% | 308 ( range 226–393) days after admission |
| Straudi | 2022 | cohort | 79 | mean : 66 | 60.8 | PHQ9 BAI | NR | NR | 100 | 42/72 | 26-week |
| Suresh | 2022 | cross-sectional study | 100 | 27:17years 43:18years 30:19years | 0 | ICD-10 | 100/1594 | NR | 45 | admitted in ICU : 3% | NA |
| Swami | 2022 | cross-sectional | 164 | mean : 47.47 | 75 | DASS-21 | NR | NR | 62 | 15.45% | 3–6 months |
| Tabacof | 2022 | cross-sectional | 156 | 44 | 31 | GAD-7 PHQ-2 | 48% | GAD-7≥10 PHQ-2≥3 | 10NA8 | NA | a median (range) time of 351 days (82–457 days) after COVID-19 infection |
| Tanriverdi | 2022 | cross-sectional | 48 | 39.2 ± 7.9 | 45.80% | Sleep quality =PSQI. HADs | NR | PSQI= 5.HADS-A=4. HADS-D=3 | 100 | NA | (after at least 12 weeks from the COVID-19 diagnosis) |
| Taquet | 2021 | cohort | 273,618 | 46.3 ±19.8 | 44.40% | ICD10 | NR | NR | NA | NA | 6months |
| Tarantino | 2022 | cross-sectional | 31 | 14.1 ± 2 | 25.8 | GAD-7 and PHQ-9 | NR | PHQ-9 ≥5, ≥10, ≥15, and ≥20 =mild, moderate, intermediate, and severe depression - GAD-7 ≥5, ≥10, and ≥15 = mild, moderate, and severe anxiety symptoms | 3 | NA | 6 months |
| Targa | 2022 | cohort | 106 | 62.0 (SD not reported) | 64.2 | PSQI | NR | PSQI > 8.0 | 100 | NA | 6 months |
| Tavares-Júnior | 2022 | cross-sectional study | 141 | mean age was 48 SD = 14 | 36.90% | Geriatric Depression Scale (GDS) was applied to assess mood, or the Beck Inventory, depending on the age of the patient | 141/207 | used a cutoff point of  3 on the GDS and 10 on the Beck inventory to diagnose depression | 35 | severe clinical condition requiring admission to icu : 3.5% | NA (diagnosis of COVID-19 confirmed in the past 12 months) |
| Titus | 2023 | cross-sectional | 1212 | ABOVE 18 | 43.9 | PHQ-2 GAD-2 | 31.8 | cut-off score of 3 + | 21 | Over 50% | 6 months |
| Titze-de-Almeida | 2022 | cohort | 362 | 41.2 ±12.8 | 39% | Anxiety (GAD-2 )/not clear | NR | GAD-2 score≥3 points | 14 | icu = 3.4% | 5–8 months after RT-qPCR |
| Tokumasu | 2022 | cohort | 279 | ME/CSF = 40 NON-ME/CSF=41 | ME/CSF = 48.9 NON-ME/CSF=43.5 | SDS EQ-5D | NR | NR | 23 | NA | more than one month (four weeks) after the onset of COVID-19 |
| Torales | 2023 | cross-sectional | 1448 | 33.9±11.2 | 26.7 | CAS | by date : 5-61% | cut-off score ≥9 | NA | NA | NA |
| van Raaij | 2022 | single-centre cohort study | 66 | median age was 60.5  (IQR: 54−69) years | 70% | GAD-7 (Generalized Anxiety Disorder scale) PHQ-9 | 66/175 | GAD-7 (cut-off score ≥10), PHQ-9 ( cut-off depression ≥ 10) | 100 | 44% | 12months |
| Vargas Ramirez | 2022 | cross-sectional | 263 | 58.0 (SD not reported) | 58.6 | Beck's anxiety and depression inventories | NR | NR | NA | NA | NA |
| Villalpando | 2022 | cross-sectional | 203 | 31.48 (SD not reported) | 35.46 | DASS-21, *no specefic scale was reported for sleep disorders | NR | DASS-21: Depression >10 and anxiety >8 | NA | NA | NA |
| Vlake | 2021 | multicenter randomized controlled trial | 89 | NA | 78% | HADS | NR | score of ≥8 (ranging from 0 to 21) | 100 | NA study population was from post-icu patients | 6months |
| Wang | 2022 | prospective cohort study | 406579 | 66.1 ±8.4 SARS-COV-2 =68.5± 8.4 control group = 68.8± 8.1 | 44.7 | ICD-10 codes | NR | NR | NA | NA | 239 days |
| Wang | 2022 | prospective cohort study | 3193 | 55.3 (sd:13.8) | corrected: 3.6% | Patient Health Questionnaire (PHQ-4)  (PHQ-2) GAD-2 | 3193/58612 | . Scores of 3 or higher on the PHQ-2 or GAD-2 indicated probable  depression or probable anxiety | NA | NA | 19 months |
| Weidman | 2021 | retrospective cohort study | 87 | median = 62 | 74 | HADS | 72.40% | greater than 8 | 100 | 100% | median time to follow-up in the recovery clinic was 20 days |
| Wiertz | 2022 | Prospective cohort study | 67 | mean = 62 | 78 | HADS | 88% | score ≥8 | 100 | 100% | 12 months |
| Xiao | 2022 | cross-sectional | 199 | 42.7 (SD not reported) | 46.7 | GAD-7 and PHQ-9 | 74.5% | cut-off point of depression is 5, cut-off point of anxiety is 5 | 100 | severe: 12.6 , critical: 9 | 6 months |
| Xu | 2022 | cross-sectional | 125 | 41.72 ± 13.61 | 57.02 | ISI and CES-D | 96.80% | ISI ≥8 and CES-D ≥16 | 100 | NA | 2 weeks |
| Yang | 2022 | prospective cohort study | 313 | 44.7 ± 17.5 | 39 | PHQ | NR | NR | 100 | 5% | 6-month and 12-month follow-up |
| Yavuz | 2022 | cross-sectional | 400 | 39.9 ± 13.7 | 39 | Beck's depression inventories (BDI) , no scale for sleep disturbences was reported | NR | NR | 24 | NA | at least one month |
| Zhu | 2020 | Multi-center retrospective cohort study | 432 | median:49 | 52.08% | Zung’s self-reported anxiety scale | NR | SAS scores of 40 or greater indicating  an unfavorable secondary outcome | 50 | 33% | NA |

**Supplementary Fig 1.** Risk of bias assessment for each included study.


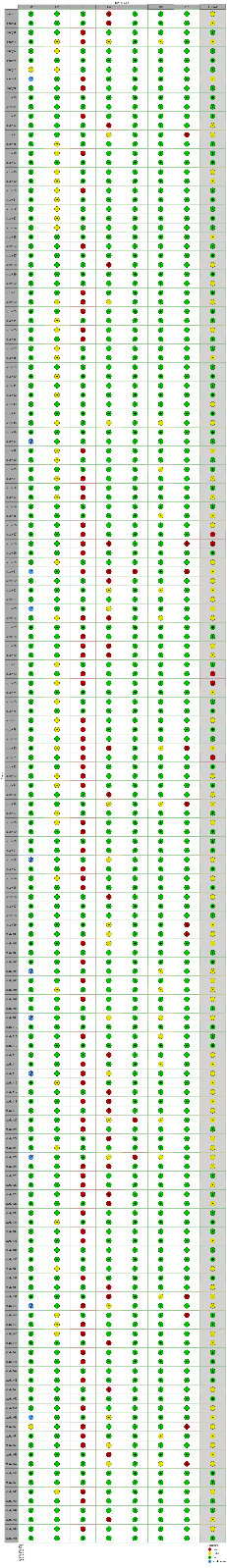


| id | author |
| --- | --- |
| 1 | Abramoff |
| 2 | Agergaard |
| 3 | Ahmed |
| 4 | Ajmi |
| 5 | Akçay |
| 6 | Albtoosh |
| 7 | Albu |
| 8 | Ali |
| 9 | Anik |
| 10 | Azevedo |
| 11 | Badinlou |
| 12 | Bahadur |
| 13 | Bai |
| 14 | Boesl |
| 15 | Boiko |
| 16 | Bonazza |
| 17 | Braga |
| 18 | brown |
| 19 | Bungenberg |
| 20 | Cacciatore |
| 21 | Cacciatore |
| 22 | Campo-Arias |
| 23 | Cansei |
| 24 | casoni |
| 25 | Chen |
| 26 | Cheung |
| 27 | Choudhry |
| 28 | Clemente |
| 29 | Clift |
| 30 | Cook |
| 31 | Dai |
| 32 | Dale |
| 33 | Damiano |
| 34 | Dankowski |
| 35 | Dar |
| 36 | Diem |
| 37 | Diem |
| 38 | Dondaine |
| 39 | Elmazny |
| 40 | Eloy |
| 41 | Ettman |
| 42 | Ezzelregal |
| 43 | Fernández-de-Las-Peñas |
| 44 | Fernández-de-Las-Peñas |
| 45 | Fernández-de-Las-Peñas |
| 46 | Fernández-de-Las-Peñas |
| 47 | Fernández-de-Las-Peñas |
| 48 | Ferrando |
| 49 | Fischer |
| 50 | Frontera |
| 51 | Fu |
| 52 | Fu |
| 53 | ghosn |
| 54 | Gorelik |
| 55 | Gramaglia |
| 56 | Grover |
| 57 | Guido |
| 58 | Guo |
| 59 | Haag |
| 60 | Hammerle |
| 61 | Harrison |
| 62 | Hartung |
| 63 | Hayek |
| 64 | Hazumi |
| 65 | Heesakkers |
| 66 | Henneghan |
| 67 | Houben-wike |
| 68 | Huang |
| 69 | Huang |
| 70 | Huang |
| 71 | Huarcaya-Victoria |
| 72 | Hüfner |
| 73 | Imran |
| 74 | Islam |
| 75 | jafri |
| 76 | jung |
| 77 | Kalamara |
| 78 | Kamran |
| 79 | Kaur |
| 80 | Kaur |
| 81 | Khatun |
| 82 | Korkut |
| 83 | Larsson |
| 84 | Lemhöfer |
| 85 | Li |
| 86 | Lier |
| 87 | Liyanage-Don |
| 88 | Magdy |
| 89 | Magnúsdóttir |
| 90 | Maley |
| 91 | Mantovani |
| 92 | Marasco |
| 93 | Matsumoto |
| 94 | Mazhari |
| 95 | Mazza |
| 96 | Mazza |
| 97 | Mazza |
| 98 | Mei |
| 99 | Mendes Paranhos |
| 100 | Méndez |
| 101 | Menges |
| 102 | Moura |
| 103 | Moy |
| 104 | Müller |
| 105 | Murga |
| 106 | Naidu |
| 107 | Naik |
| 108 | Nehme |
| 109 | Niedziela |
| 110 | Noviello |
| 111 | Ocsovszky |
| 112 | Pappa |
| 113 | Park |
| 114 | Pasin |
| 115 | Patel |
| 116 | Pathak |
| 117 | Pellitteri |
| 118 | Peluso |
| 119 | Perrot |
| 120 | Pistarini |
| 121 | Pudlo |
| 122 | Rass |
| 123 | Richter |
| 124 | Richter |
| 125 | Rousseau |
| 126 | Salehian |
| 127 | Salimi |
| 128 | Santos |
| 129 | Sathyamurthy |
| 130 | Sayde |
| 131 | Schneider |
| 132 | Schulze |
| 133 | Senara |
| 134 | Shah |
| 135 | Shukla |
| 136 | Singh |
| 137 | Speichert |
| 138 | Staudt |
| 139 | Straudi |
| 140 | Suresh |
| 141 | Swami |
| 142 | Tabacof |
| 143 | Tanriverdi |
| 144 | Taquet |
| 145 | Tarantino |
| 146 | Targa |
| 147 | Tavares |
| 148 | Titus |
| 149 | Titze |
| 150 | Tokumasu |
| 151 | Torales |
| 152 | van |
| 153 | Vargas |
| 154 | Villalpando |
| 155 | Vlake |
| 156 | Vlake |
| 157 | Wang |
| 158 | Wang |
| 159 | Weidman |
| 160 | Wiertz |
| 161 | Xiao |
| 162 | Xu |
| 163 | Yang |
| 164 | Yavuz |
| 165 | zhu |
